# A Novel HPLC-MS/MS Method for the Intracellular Quantification of the Active Triphosphate Metabolite of Remdesivir: GS-443902

**DOI:** 10.3390/jox15040107

**Published:** 2025-07-03

**Authors:** Alice Palermiti, Amedeo De Nicolò, Miriam Antonucci, Sara Soloperto, Martina Billi, Alessandra Manca, Jessica Cusato, Giorgia Menegatti, Mohammed Lamorde, Andrea Calcagno, Catriona Waitt, Antonio D’Avolio

**Affiliations:** 1 Laboratory of Clinical Pharmacology and Pharmacogenetics, Department of Medical Sciences, University of Turin, Amedeo di Savoia Hospital, 10149 Turin, Italy; 2 ASL Città di Torino, Amedeo di Savoia Hospital, 10149 Turin, Italy; 3 Infectious Diseases Institute, Makerere University College of Health Sciences, Kampala P.O. Box 22418, Uganda; 4 Unit of Infectious Diseases, Department of Medical Sciences, University of Turin, 10149 Turin, Italy; 5 Department of Pharmacology and Therapeutics, University of Liverpool, Liverpool L69 7BE, UK

**Keywords:** pharmacokinetics, COVID-19, chromatography, mass spectrometry, peripheral blood mononuclear cells

## Abstract

Background: Remdesivir (RDV) is a broad-spectrum antiviral prodrug, which is rapidly metabolized in vivo within cells to the pharmacologically active triphosphate metabolite, GS-443902. On the other hand, the dephosphorylated metabolite GS-441524 is the main form detected in plasma. RDV acts against RNA viruses, and it was the first antiviral drug to receive EMA and FDA approval for treating COVID-19. Nevertheless, its intracellular pharmacokinetics in real life are poorly explored, particularly due to technical challenges. Methods: The aim of this study was to validate an HPLC-MS/MS method for the direct quantification of GS-443902 in peripheral blood mononuclear cells (PBMCs) with a chromatographic separation of 15 min. Results: The method was validated following EMA and FDA guidelines in terms of sensitivity, specificity, accuracy, precision, matrix effect, recovery, carryover, and stability, and then applied to PBMC isolates from a small cohort of patients with severe COVID-19 who received RDV. Conclusions: This work represents the first method for the direct quantification of GS-443902 in PBMCs, with possible future application to intracellular pharmacokinetic studies in different scenarios, such as new oral prodrugs or drug–drug interaction studies.

## 1. Introduction

Beginning in December 2019, an outbreak of severe acute respiratory syndrome coronavirus type 2 (SARS-CoV-2) rapidly spread throughout the world and was officially declared a pandemic by the World Health Organization (WHO) on 11 March 2020 [1,2]. At the onset of the COVID-19 pandemic, no specific antiviral treatment was available, and patients primarily received supportive care to manage symptoms [3]. During the initial waves, the strategy of repurposing existing drugs with diverse therapeutic uses against SARS-CoV-2 emerged as a key approach in the effort to develop effective treatments [4]. Among these, remdesivir (RDV, formerly known as GS-5734), a prodrug of the adenosine analogue GS-441524, demonstrated high in vitro activity against SARS-CoV-2 and showed clinical effectiveness, especially when administered early in the course of infection [5]. RDV is a broad-spectrum antiviral agent, with activity against a variety of RNA viruses, including filoviruses, pneumoviruses, paramyxoviruses, and coronaviruses [6,7,8,9,10].

**Figure 1:**
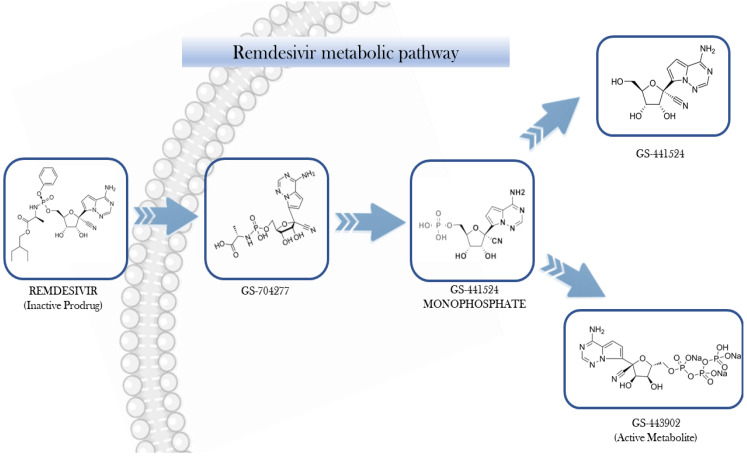
Step-wise bioactivation of remdesivir.

After intravenous administration, RDV undergoes intracellular conversion into its active form, the nucleoside triphosphate analogue GS-441524-TP (also known as GS-443902). Meanwhile, its nucleoside form, GS-441524, produced through dephosphorylation, can be detected in the plasma (Figure 1) [11].

GS-443902 exhibits high polarity, limiting its capacity to permeate cellular membranes directly. In contrast, its prodrug, RDV, is capable of cellular entry predominantly through passive diffusion and potentially via solute carrier (SLC) transporters. The pharmacokinetics (PKs) of RDV may be modulated by various transport mechanisms, including uptake transporters such as organic anion transporting polypeptides (OATP1B1and OATP1B3B3), concentrative nucleoside transporters (CNTs), and equilibrative nucleoside transporters (ENTs), as well as efflux proteins like multidrug resistance-associated protein 4 (MRP4) and P-glycoprotein (P-gp). Once internalized, RDV undergoes a series of enzymatic transformations involving carboxylesterase 1 (CES1), cathepsin A, histidine triad nucleotide-binding protein 1 (HINT1), and cellular kinases, ultimately yielding the active metabolite GS-443902. The intracellular concentration of GS-443902 is markedly influenced by the tissue-specific expression of these metabolic enzymes and transporters [12,13].

The metabolic activation of RDV is initiated by the esterase-mediated hydrolysis, yielding the intermediate metabolite GS-704277. Subsequent cleavage of the phosphoramidate group enables phosphorylation steps that ultimately produce GS-443902, the pharmacologically active triphosphate. This active metabolite selectively targets viral RNA-dependent RNA polymerase, without interfering with host RNA or DNA polymerases [9,14]. From a mechanistic perspective, GS-443902 competes with adenosine triphosphate (ATP) for incorporation into newly synthesized viral RNA, leading to delayed termination of the RNA chain. [15,16,17].

From a PK standpoint, RDV demonstrates significantly greater plasma protein binding compared to its nucleoside analog GS-441524 (approximately 80–90% versus >20%). RDV undergoes extensive hepatic metabolism via cytochrome P450 isoenzymes (CYP2C8, CYP2D6, and CYP3A4), accounting for its low oral bioavailability due to the pronounced first-pass effect [18,19]. The estimated mean elimination half-lives are 0.89 h for RDV and 25 hours for GS-441524, respectively [19]. Urinary excretion constitutes the primary elimination route, with approximately 74% of the administered dose recovered, predominantly as GS-441524 [20]. While RDV, GS-704277, and GS-441524 are measurable in plasma, GS-443902 is detectable exclusively within cells [21]. In clinical practice, RDV is indicated for adult and adolescent patients with COVID-19 pneumonia requiring supplemental oxygen (low- or high-flow, or non-invasive ventilation), and for adults at elevated risk of progression to severe disease despite not requiring oxygen support [5].

Although both RDV and GS-441524 exhibit in vitro activity against SARS-CoV-2, their antiviral efficacy is directly correlated with intracellular concentrations of GS-443902 [18]. However, current PK data regarding intracellular GS-443902 levels still remain poorly explored in the literature.

A recent investigation reported high intracellular concentrations of GS-443902 in peripheral blood mononuclear cells (PBMCs) from RDV-treated individuals, supporting the use of PBMCs as a surrogate model for assessing intracellular drug [18].

Notably, in that study, GS-443902 was quantified indirectly via enzymatic dephosphorylation followed by GS-441524 measurement; this kind of approach is cumbersome and does not allow distinguishing between mono-, di-, and triphosphate forms.

In the present work, we describe the development, validation (in accordance with EMA and FDA regulatory standards), and clinical application of a high-performance liquid chromatography coupled with tandem mass spectrometry (HPLC-MS/MS) method for the direct quantification of intracellular GS-443902 in PBMCs derived from COVID-19 patients undergoing RDV therapy.

## 2. Materials and Methods

### 2.1. Chemicals and Reagents

HPLC-grade acetonitrile (ACN) and methanol (MeOH) were purchased from VWR Chemicals (Radnor, PA, USA); MS-grade H_2_O (MilliQ) was produced using a Milli-DI system coupled with a Synergy 185 system by Millipore (Milan, IT, Italy); dimethyl sulfoxide (DMSO), diethylamine, and exylamine were purchased from the Sigma–Aldrich Corporation (Milan, IT, Italy). Acetic acid was purchased from TitolChimica (Rovigo, IT, Italy).

^2^H_6_-tenofovir diphosphate (^2^H_6_-TDF-DP) was employed as the internal standard (IS) and was purchased from Toronto Research Chemicals (TRC), Canada. Blank buffy coats from healthy donors were provided by the Blood Bank of “Città della Salute e della Scienza” of Turin (IT, Italy). According to HPLC analysis, each compound had a purity greater than 95%. RDV (purity 99%), its metabolite GS-441524 (purity 99.1%), and its triphosphate metabolite triethylammonium salt (GS-443902, purity 99.8%) were purchased from AlsaChim, France. All powders were stored at −20 °C in the dark.

### 2.2. Standards and Internal Quality Control Samples

A stock solution of GS-443902 was prepared at a concentration of 1 mg/mL and stored in the dark at −80 °C until use. To prepare the highest standard (STD 9) and the Quality Control (QC) samples, a concentrated working solution was made by diluting the stock solution with a 50:50 mixture of MeOH and H_2_O. This solution was also stored at −80 °C. Lower concentration standards (STD 1–8) were prepared through serial 1:1 dilution starting from the highest standard sample. For each analysis session, 100 µL of the standard solutions were added to PBMC samples (10 million cells in 0.5 mL) to prepare the matrix-matched calibration points and quality controls. Exact concentrations for each standard (STD), calibration ranges, and QC values are detailed in Table 1.

### 2.3. PBMCs Isolation

Adult patients with severe COVID-19 who were treated with remdesivir (RDV) and provided written informed consent were enrolled in this study. The study was conducted in accordance with the Declaration of Helsinki and local institutional review board regulations, following approval from the local Ethics Committee (“A.O.U. Città della Salute e della Scienza di Torino—A.O. Ordine Mauriziano di Torino—A.S.L. Città di Torino”, Protocol E-COVID, No. 00171/2020). Blood samples were collected using lithium heparin tubes (for plasma) and cell preparation tubes (CPT^®^, Becton, Dickinson and Co., Franklin Lakes, NJ, USA) for PBMC isolation. Plasma was obtained by centrifugation at 1400× *g* for 10 min at 4 °C (Jouan Centrifuge, Model BR4i, Saint-Herblain, France).

PBMC isolation from blood samples (2 × 8 mL CPT, 16 mL total volume) was performed following a previously described protocol [22,23].

After PBMC isolation, cell counting and mean cell volume (MCV) were performed for each sample using an automated Z2 Beckman Coulter (Instrumentation Laboratory, Milan, Italy). Cell pellets were resuspended in 1 mL of H_2_O–MeOH (30:70, *v*/*v*), divided into two 500 μL aliquots, and stored at −80 °C until analysis. Sample-specific cell counts and mean corpuscular volumes (MCVs) were used to calculate the total cell volume in each PBMC aliquot, thereby allowing conversion of concentrations from ‘ng/aliquot’ to ‘ng/mL’, as previously described [22,23].

Drug-free PBMCs used for the preparation of standards (STDs) and QCs were isolated using the same protocol from buffy coats from healthy donors.

Before each analytical session, each blank PBMCs aliquot was diluted with H_2_O–MeOH 30:70 (*v*/*v*) in order to obtain a fixed concentration of 10 × 10^6^ cells for calibrators. For pharmacokinetic sampling timepoints, C_trough,_ C_max_, and 1 h post-infusion were selected, due to their relevance for capturing key phases of intracellular drug exposure [24]. The C_max_ reflects the expected peak plasma concentration of RDV and its metabolites, typically occurring shortly after the end of the infusion, which is crucial for evaluating the extent of cellular uptake. Since this interval has been shown to reflect active intracellular phosphorylation processes, the 1 h post-infusion time point was included to assess the early intracellular disposition of GS-443902 [17,25]. The C_trough_, or pre-dose level at the steady-state, provides essential information regarding the residual intracellular drug concentration before the next dosing cycle and is relevant for evaluating drug accumulation and steady-state kinetics [26].

### 2.4. Sample Preparation

The intracellular extraction procedure involved cell lysis and sample purification, as described in previous works [27,28]. All the extracted samples were subsequently analyzed using HPLC-MS/MS. An internal standard (IS) working solution in MeOH–H_2_O (70:30 vol/vol) was prepared at a concentration of 100 ng/mL for each session. After thawing at room temperature, 100 µL of calibration standard (STD) and 50 µL of the IS working solution were added to 500 µL of the PBMCs aliquot.h

Real samples from patients underwent the same procedure: 50 µL of IS working solution and 100 µL of MeOH: H_2_O (50:50 vol/vol, to mimic the spike with the calibrating solutions) were added to the patient cell aliquot of 500 µL, thereby obtaining the same volume and composition.

Then, all the samples were vortex-mixed for at least 10 s and sonicated for 5 min without heat.

After, samples underwent centrifugation at 21,000× *g* for 10 min at 4 °C and supernatants were dried in a vacuum centrifuge at 40 °C for about 1.5 h, in glass bacteriological tubes. Extracts were finally reconstituted using 100 µL of a mixture of 85% mobile phase A (composed by H_2_O with 5mMol hexylamine, 0.4% dimethylamine and 2 mL of mass grade acetic acid), and 15 % of phase B, (composed by ACN mass grade and phase A 60:40 (*v*/*v*)).

After resuspension, 20 µL from each vial was injected for chromatographic analysis.

### 2.5. HPLC-MS/MS Settings

Chromatographic analysis was performed using a Perkin Elmer LX-50 UHPLC system coupled with a QSight 220 Triple Quadrupole mass spectrometer (Perkin Elmer, Milan, Italy). The autosampler temperature was maintained at 15 °C. HPLC separation was performed using a Hypercarb^®^ 150 mm × 2.1 mm column (ThermoScientific, Waltham, MA, USA), with a gradient run of 2 mobile phases (A and B) at 35 °C, at 0.4 mL/min.

Mobile phase A was composed of H_2_O with 5mMol hexylamine, 0.4% diethylamine, and 2 mL of mass grade acetic acid, while mobile phase B was composed of ACN mass grade and phase A, 60:40 vol/vol. The initial condition was 85% Mobile Phase A held up to 1 min, then it decreased gradually to 70% at 2.8 min and this percentage was kept up to 6 min; then, mobile phase A was set at 20% for 4 min (up to 12 min) and, finally, re-equilibrated for 4 min at the initial mobile phases composition.

The total runtime was 16 min. Two strong washing (H_2_O–ACN 30:70 *v*/*v*) and two weak washing (H_2_O:MeOH 95:5 *v*/*v*) steps (250 µL each) were performed after the injection of each sample.

The mass spectrometric detection was operated with an electrospray ionization (ESI) interface and negative ionization (ESI-).

Nebulizing and heating gas was “Zero-Air” (Dry air), while drying and collision gas was nitrogen; both these gases were produced at high purity (>99.9%) with a Cinel Zefiro QS^®^ (Cinel, Vigonza, Italy).

Two highly sensitive mass transitions were selected for both the analyte and the IS, one used for quantification and the other one for qualification. These are reported, together with the general source parameters, in Table 2.

### 2.6. Method Validation

Once optimal separation of the analytes was achieved, the method underwent full validation in accordance with EMA and FDA guidelines for bioanalytical method validation [29]. The validation process assessed key performance parameters, including specificity, sensitivity, accuracy, precision, linearity, matrix effects, recovery, and carry-over. In addition, both short-term and long-term stability studies were conducted.

#### 2.6.1. Specificity and Selectivity

Specificity and selectivity were evaluated on 10 analyte-free samples for each tissue, undergoing the same processing protocol. The absence of interfering peaks with areas exceeding 20% of the target analyte’s peak at the Lower Limit of Quantification (LLOQ), and 5% of the internal standard (IS) peak at their respective retention times, was considered indicative of good specificity.

#### 2.6.2. Accuracy, Precision, Calibration, Sensitivity, Dilution Integrity

Accuracy and repeatability (intra-day precision) were evaluated by calculating the coefficient of variation (CV%) across five replicates at each quality control (QC) concentration level. Reproducibility (inter-day precision) was assessed by determining the CV% of QCs across six independent validation sessions. Calibration curve linearity was also assessed over six validation sessions.

Sensitivity was expressed in terms of the Lower Limit of Quantification (LLOQ) and the Limit of Detection (LOD), defined as the lowest concentrations yielding a signal-to-noise ratio of 10 and 3, respectively. Additionally, both the percentage deviation from the nominal concentration (bias%) and the CV% at the LLOQ level (evaluated in the same manner as other QC levels) were required to be below 20%. Dilution integrity was assessed using duplicate samples with concentrations twice that of the highest calibration standard (STD 9), following a 3-fold dilution.

#### 2.6.3. Recovery

Recovery (REC) was assessed by comparing the peak areas of the analyte and internal standard (IS) in six analyte-free PBMC samples from different donors, spiked with the expected QC concentrations after the extraction process (post-extraction spiking), to those obtained from QC samples spiked before extraction (pre-extraction spiking). This comparison allowed the estimation of the percentage recovery under the assumption of 100% recovery in post-extraction spiked samples. In order to test IS performance in limiting the variability in REC, this parameter was evaluated both as “absolute “and “IS-normalized”.

#### 2.6.4. Adsorption and Stability

Preliminary adsorption tests on plastic and glass vials were conducted by injecting GS-443902 at low, medium, and high concentrations in pure solvents from plastic and LC-MS certified glass vials. Long-term storage stability was evaluated up to 3 months at −80 °C by comparison of freshly prepared QCs and stored QCs. Short-term stability was evaluated bench-top at room temperature (25 °C) and artificial light up to 12 h, by comparison with immediately thawed and analyzed QCs, and in the autosampler up to 24 h after extraction, by comparison with freshly extracted QCs.

Freezing and thawing stability was investigated up to two “freeze and thaw” cycles by comparing with freshly prepared QCs.

#### 2.6.5. Matrix Effect

ME percentage was evaluated by comparing the peak areas of the analyte and IS in “post-extraction” spiked samples (as reported in Section 2.6.3) containing matrix components, with the same peak areas from the injection of solvents spiked at the same concentrations of analyte and IS [30]. Aliquots with different numbers of cells were used for this purpose, at 5, 10, and 20 million cells/aliquot.

#### 2.6.6. Carry-Over

Carry-over was assessed by analyzing analyte- and IS-free plasma extracts immediately following the injection of samples containing analyte and IS concentrations twice those of the highest calibration standard (STD 9).

Carry-over was defined as acceptable if the signal in these samples was lower than 20% of the LLOQ signal for the analyte and 5% for the IS.

#### 2.6.7. Application and Statistical Analysis

The analytical method was applied to a preliminary analysis of 15 PBMC extracts collected from five COVID-19 patients enrolled in the e-COVID study, at three pharmacokinetic time points: prior to RDV infusion (C_trough_), at the end of infusion (C_max_), and one hour post-infusion (C_1h_). The aim was to assess the method’s suitability for pharmacokinetic studies. Chromatographic data were processed using Simplicity^®^ 3Q software (Perkin Elmer, Milan, Italy). Concentration values were reported as means with 90% confidence intervals, calculated using SPSS version 29.0 (IBM, Armonk, NY, USA). For comparative purposes, RDV and GS-441524 plasma concentrations were determined using a previously validated UHPLC–MS/MS method [31].

#### 2.6.8. Incurred Sample Reanalysis

Samples were tested in 2 independent analytical sessions. The resulting CV% was considered as a measure of “incurred samples” precision.

## 3. Results

### 3.1. Calibration Curve Linearity and Dilution Integrity

During method validation sessions, the coefficients of determination (R^2^) for the calibration curves ranged from 0.996 to 0.999, demonstrating an excellent fit to the linear model with 1/conc weighting.

Quantification of samples spiked with concentrations exceeding the highest calibrator standard (STD 9) resulted in a mean bias of less than 10% following a three-fold dilution with blank sample matrix prior to extraction, thereby demonstrating acceptable dilution integrity.

The average slope of the standard curve was 0.1657 (RSD = 6.2%), and the mean intercept was 0.0021 (RSD = 21.3%).

### 3.2. Specificity and Selectivity

**Figure 2:**
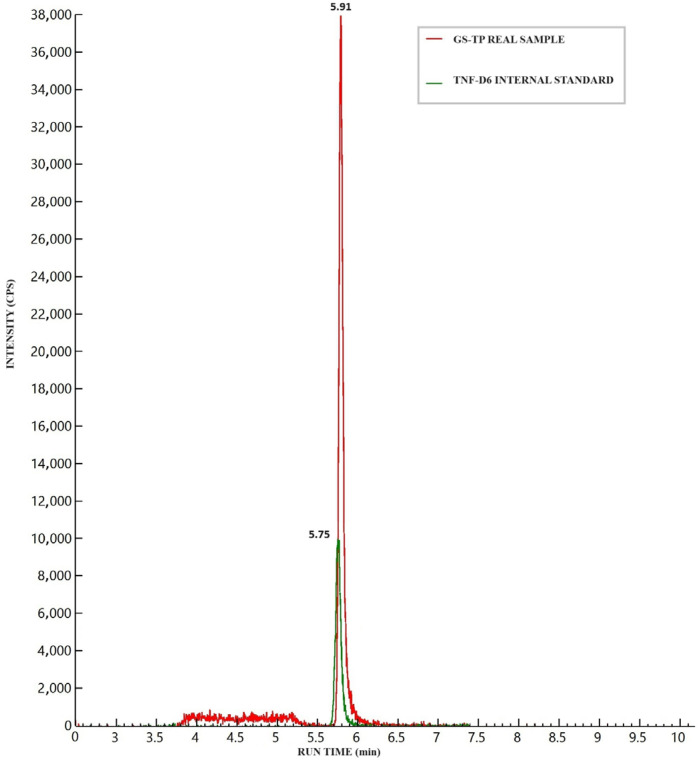
Chromatographic peaks of GS-443902 (analyte) and ^2^H_6_-TFD-DP (internal standard, IS) after the injection of a medium quality control (16 ng/sample) prepared in a 20 million cell PBMC aliquot. The isobaric interference from matrix components can be seen between 3.8 and 5.3 min, well separated from the analyte’s retention time.

Figure 2 illustrates the chromatographic separation of GS-443902, IS, and ^2^H_6-_TFD-DP in a medium Quality Control (QC) sample (16 ng) spiked into an aliquot containing 20 million cells. No significant background signal (“noise”) was observed in blank cell samples at the analyte retention time (RT), which was defined as 20% of the analyte signal at the LLOQ or 5% of the IS signal. Although a notable signal from isobaric endogenous matrix components, proportional to the number of cells, was detected, it was adequately resolved during the chromatographic run (see Figure 2).

### 3.3. LLOQ and LOD

The observed LOD was 0.104 ng/sample, while the LLOQ was at least equivalent to that of the lowest calibrator standard (STD1) (0.313 ng/sample), in accordance with FDA guidelines. Chromatograms for a blank (analyte-free) sample and LLOQ are presented in Figure S1. The LLOQ accuracy and precision, as assessed in quality control samples, were 96% and 95% respectively.

### 3.4. Adsorption and Stability

Preliminary adsorption tests on plastic and glass surfaces were conducted and demonstrated no significant differences in compound stability and signal intensity between glass and plastic (polypropylene) vials, indicating negligible adsorption. More concentrated stock solutions were stored in borosilicate glass vials.

**Figure 3:**
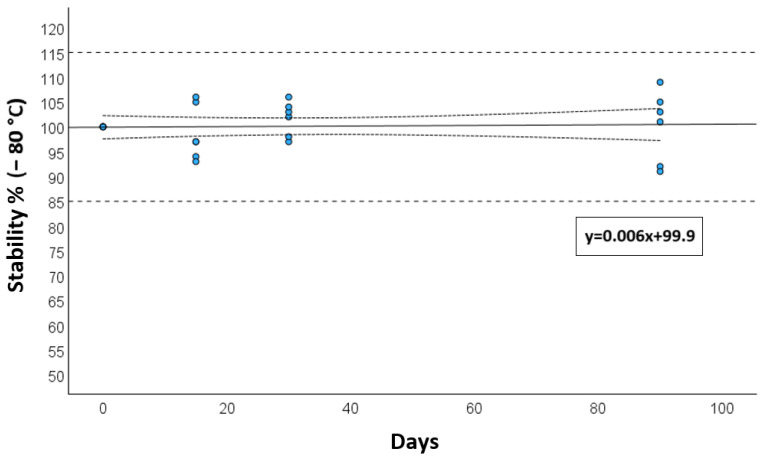
Long-term stability at −80 °C for calibrators and quality controls after 15, 30 days, and 3 months. The solid reference line is the mean degradation curve, the dotted lines represent the 95 CI for the degradation curve, while the dashed lines show the range of acceptance of stability (±15%).

Long-term storage stability, assessed at −80 °C, showed a deviation of less than 15% after 3 months, as presented in Figure 3.

Similarly, short-term stability under bench-top conditions and up to two freeze and thaw cycles was found to be satisfactory for up to 24 h, with a mean degradation of 3.5%. Regarding autosampler stability, a decrease in signal was observed after one day, occurring at a comparable rate for both the analyte and the IS. As a result, accurate quantification was maintained as the analyte/IS ratio remained consistent across all QC concentrations tested (RSD = 3.9%).

### 3.5. Recovery and Matrix Effect

Recovery (REC) data, both in terms of absolute REC and IS-normalized REC, were consistent and highly reproducible for each analyte. Similarly, the matrix effect (ME) was observed, exhibiting considerable variability across different cell matrices. However, the evaluation of IS-normalized ME (IS-nME) demonstrated the strong performance of the selected IS compounds in compensating for the variability introduced by ME. These findings are consistent with previous studies and comply with EMA guidelines [29,30,32]. The data are summarized in Table 3. Moreover, the percentage of accuracy and precision calculated for QCs samples was 93% and 96%, respectively.

### 3.6. Carry-Over

Carry-over investigations revealed the absence of significant peaks, with values lower than 10% of the LLOQ for RDV and 1% for IS.

### 3.7. Testing of Patients’ Samples

The presented method was applied to 15 PBMC extracts from 5 patients at 3 time points, all receiving a standard daily intravenous infusion of 100 mg of RDV. GS-443902 was successfully quantified in each sample, with the lowest observed amount in a single PBMCs aliquot being 3.16. Table S1 summarizes the coupled concentrations of GS-443902 in PBMC alongside the plasma concentrations of GS-441524 and RDV (the prodrug).

The mean concentrations (CI90) of intracellular GS-443902 were as follows: 4853 ng/mL (2232–7474) at C_trough_, 10,735 ng/mL (5802–15,668) at C_max_, and 9140 ng/mL (5164–13,116) 1 h after the end of infusion. Reanalysis of incurred samples demonstrated acceptable reproducibility, in compliance with EMA and FDA guidelines (RSD < 10%).

## 4. Discussion

The antiviral activity of RDV is understood to be mediated by its intracellular active metabolite, GS-443902.

In this work, we described a validated method for the direct and specific quantification of GS-443902 in PBMCs, used as a surrogate cell type for assessing drug uptake and phosphorylation. This approach offers several advantages. It does not require enzymatic dephosphorylation, which is cumbersome and incapable of discriminating between mono-, di-, or tri-phosphate metabolites [33]. It is also superior to anion exchange solid phase extraction (SPE), which is expensive, complex, and only partially specific in discrimination between differently phosphorylated forms [34].

The evaluation of RDV concentrations at the intracellular level could provide valuable insight into its cellular uptake, thereby enabling more detailed drug concentration data in patients across various clinical scenarios, such as the presence of potential drug–drug interactions, the use of different formulations, doses, or in patients with varying genetic backgrounds.

The primary limitation to the clinical use of RDV is its requirement for intravenous infusion, due to its low oral bioavailability. This limitation has prompted extensive research into the development of alternative oral prodrugs capable of generating the same intracellular active metabolites or the potential use of GS-441524 itself as an oral prodrug [35].

In this context, the oral prodrugs mindeudesivir and obeldesivir have recently gained attention due to their promising results in both pre-clinical and clinical trials, demonstrating superiority over placebo and non-inferiority against nirmatrelvir-ritonavir [1,35,36]. Since these drugs produce the same active intracellular triphosphate metabolite, GS-443902, its quantification within cells represents the only valid method for comparing their theoretical antiviral efficacy. A similar situation was observed when investigating the switch between different prodrugs, such as tenofovir disoproxil fumarate (TDF) and Tenofovir alafenamide (TAF) [37]; in this scenario, the presented method could be highly useful. A minor concern may arise regarding the mono-deuteration in the GS-441524 (and consequently GS-443902) in mindeudesivir. Nevertheless, this difference is not expected to significantly influence its retention time in this type of HPLC method, particularly considering the shared ion-coupling mechanics. Similarly, from an MS/MS perspective, the ionization efficiency and the Multiple Reactions Monitoring (MRM) transitions are also expected to be identical, requiring only the addition of 1 Dalton to the monitored m/z, while leaving all other parameters unchanged.

Using PBMCs as a surrogate for lung tissue in this study could present biological limitations. While PBMCs are a commonly employed and accessible cell type for PK analysis, they do not fully replicate the characteristics of the target cells in the lungs, such as pneumocytes. The pharmacokinetic behavior of drugs can differ substantially across tissues due to differences in cell composition, metabolic activity, and drug transport mechanisms. Consequently, while PBMCs offer valuable insights, they may not fully replicate the drug concentrations or pharmacological effects in pulmonary cells, and further research using lung tissue or more representative models is essential to confirm the relevance of these findings to target tissues in diseases like COVID-19.

One notable limitation of this study is the relatively small sample size, which inherently limits the generalizability of our pharmacokinetic findings. While the data provide valuable preliminary insights into the intracellular behavior of GS-443902 in PBMCs, the limited number of subjects may not fully capture the variability present in a broader population. Therefore, caution should be exercised when extrapolating these results to other clinical settings or patient groups. Future studies involving larger cohorts are warranted to confirm these findings and strengthen the robustness of the described pharmacokinetic profile. Another significant challenge in the development of this method was evaluating its validity in a range of cell numbers and different matrix lots, as intracellular concentrations of matrix components with ion-suppression potential are expected to be high.

For this reason, the evaluation of ME and REC was conducted using six different PBMC batches at three different cell concentrations (5, 10, and 20 million cells) from different healthy donors.

Both IS-nREC and IS-nME yielded satisfactory results using both the “standard curve slopes” and the “post-extraction addition” methods. It is worth noting that absolute REC and ME varied between samples, underscoring the crucial role of the IS in this type of analysis.

Nevertheless, although the IS-nREC at the QC H levels remain borderline (Table 3), the ICH guidelines emphasize the importance of demonstrating that matrix effects do not compromise the specificity and reliability of the analytical method. In this method, the matrix effect was thoroughly assessed and managed to ensure accurate and reproducible results [29]. Unfortunately, at the time of this study, a stable isotope-labeled IS for GS-443902 was not available. Therefore, ^2^H_6_-TFD-DP served as an ideal alternative, as it shares several chemical properties, including being an ATP analogue with similar potential for ion coupling, pKa, and molecular mass. Another consideration, beyond the potential for ion suppression, was the observed interference from isobaric matrix components that shared the same mass transitions as GS-443902. These components may include other endogenous triphosphate nucleosides.

However, this method provided sufficient separation of these interfering molecules, enabling robust quantification of the target compound in up to 20 million cells.

The matrix effect observed across different PBMC batches represents a potential source of variability that could impact the reproducibility of the method in inter-laboratory settings. The variability data for ME and REC, account for the potential error when the calibration is performed in matrix, with CVs being less than 15% (Table 3) Although IS normalization was applied to minimize this impact, it does not entirely eliminate batch-related differences, highlighting the importance of considering matrix-related variability in future studies using similar approaches.

The direct application of this method to samples from patients undergoing anti-SARS-CoV-2 treatment at a dose of 100 mg demonstrated that all the analyzed samples were well above the LLOQ. These results confirmed the method’s ability to quantify intracellular concentrations in human samples, across a wide range of drug levels (from a minimum of 1200 to 15,000 ng/mL) and were in close agreement with previous reports in humans, as detailed by Humeniuk et al. [18]. The observed trough concentration, expressed as molarity, was 9.1 µM (CI_90_ 4.21–14.10), which closely aligns with the 10.2 µM reported by Humeniuk et al. in healthy individuals. However, it is important to note that this exploratory evaluation was conducted after 5 to 7 days of treatment, representing the steady state concentration for GS-441524 in plasma, but not yet for GS-443902, which has a longer half-life (25 h vs. 46 h) [18].

Moreover, in accordance with EMA guidelines, the incurred sample reanalysis demonstrated satisfactory reproducibility (CV < 15%), confirming the reliability of sample testing across multiple analytical sessions. Additionally, stability data indicated sufficient short- and long-term stability under our working conditions.

Finally, while the current discussion briefly addresses the limitations of indirect GS-443902 quantification methods, a more comprehensive comparison with previously published techniques is necessary to fully highlight the advantages and novelty of the present method. Most published approaches quantify GS-443902 indirectly by measuring its precursor, GS-441524, or through enzymatic dephosphorylation of intracellular triphosphates, followed by surrogate analyte detection [21,31]. Although these techniques are useful, they introduce additional sample processing steps, which can compromise analyte stability, introduce enzymatic variability, and limit quantification accuracy, particularly at lower concentrations.

Indirect quantification methods also depend significantly on assumptions regarding enzymatic efficiency and conversion rates, which may not accurately reflect the true intracellular levels of the pharmacologically active triphosphate. In contrast, our method directly quantifies GS-443902 within PBMCs using a validated HPLC-MS/MS protocol, providing enhanced specificity and minimizing sample manipulation. This direct detection improves reliability, reduces loss or degradation during sample preparation, and enables a more accurate assessment of the intracellular pharmacokinetic profile [21].

To the best of our knowledge, few studies have utilized a similarly direct approach with the sensitivity and robustness demonstrated in this work. By eliminating the need for enzymatic conversion or chemical dephosphorylation, our method offers a streamlined and reproducible workflow that is easily adaptable for clinical pharmacokinetic studies. We believe that these methodological advances not only enhance quantification accuracy but also provide a valuable tool for future research investigating intracellular drug dynamics across different cell types or treatment regimens [18,38].

Nevertheless, the direct and specific quantification of GS-443902 presents some limitations: particularly the relatively long runtime (15 min) due to extended equilibration time associated with ion coupling and the use of a graphitic carbon column, which is only available in HPLC format (3 µm). Furthermore, PBMCs are not pneumocytes; therefore, in the context of COVID-19, they should be considered surrogate cellular models for RDV intracellular penetration and activation to the active triphosphate metabolite.

It is important to emphasize that RDV, GS-441524, and other oral prodrugs such as obeldesivir or mindeudesivir are broad-spectrum antivirals, with potential applications across a variety of viral infections and targeting a broad range of cell types. For example, the intracellular quantification of GS-443902 in PBMCs is expected to yield valuable insights into infections that primarily affect lymphocytes or monocytes/macrophages, such as those caused by ebolavirus [39].

## 5. Conclusions

This is the first validated method reported for the direct quantification of RDV metabolite GS-443902 in PBMCs.

This approach enables the assessment of intracellular concentrations in nanograms per milliliter (ng/mL), facilitating comparison with plasma concentrations, the standard metric for therapeutic drug monitoring. Notably, the quantification of RDV triphosphate in PBMCs provides valuable insights into drug penetration at the cellular level, potentially influencing its pharmacological activity. Consequently, this method holds significant potential and broader application in future clinical studies involving RDV or other prodrug candidates.

## Figures and Tables

**Table 1:** GS-443902 concentration in the calibrating solution [ng/mL] and GS-443902 amount in the PBMC sample [ng] for calibrators and quality control levels. Standard (STD); Quality Control (QC); High (H); Medium (M); Low (L).

	GS-443902 Concentration in the Calibrating Solution [ng/mL]	GS-443902 Amount in the PBMC Sample [ng]
STD 1	3.125	0.3125
STD 2	6.25	0.625
STD 3	12.5	1.25
STD 4	25	2.5
STD 5	50	5
STD 6	100	10
STD 7	200	20
STD 8	400	40
STD 9	800	80
QC H	640	64
QC M	160	16
QC L	40	4

**Table 2:** General detector settings and analyte-specific parameters. HSID, Heated Surface Induced Desolvation; RF, Radio Frequency; ESI, Electrospray ionization mode.

General Detector Settings				
Drying Gas Temperature [°C]				130
HSID Temperature [°C]				300
Nebulizer Gas [L/h]				350
ElectroSpray V1 Negative [kV]				−4.8
Source Temperature [°C]				350
Multipole 1 RF				370
Collision Pressure				420
Ionization				ESI-
Analyte-Specific Parameters				
	GS-443902		2H6-TNF-DP (IS)	
	Primary	Secondary	Primary	Secondary
Ion Trace (m/z)	529.9 > 158.9	529.9 > 431.9	452.0 > 176.9	452.0 > 354.9
Collision Energy	43	30	31	28
Entrance voltage	−29	−39	−23	−23
Collision Cell Lens 2	100	120	96	92

**Table 3:** Summary of validation parameters by analyte concentration. IS-nREC = Recovery normalized by Internal Standard; IS-nME = Matrix Effect normalized by Internal Standard; IS-nEE = Extraction Efficiency normalized by Internal Standard.

		Recovery Mean% (RSD%)	Matrix Effect Mean% (RSD%)	Extraction Efficiency Mean% (RSD%)	IS-nREC Mean% (RSD%)	IS-nME Mean% (RSD%)	IS-nEE Mean% (RSD%)
GS-443902	H	51.2 (21.3)	53.7 (17.)	95.0 (8.1)	119.0 (3.7)	113.3 (7.1)	101.2 (5.1)
	M	85.8 (14.6)	67.3 (15.4)	101.9 (11.2)	114.0 (11.4)	112.9 (9.2)	98.8 (1.7)
	L	119.5 (13.7)	114.6 (15.3)	105.8 (18.2)	114.0 (3.4)	111.7 (11.9)	98.9 (2.9)

## Data Availability

The data that support the findings of this study are available on request from the corresponding author, A.D.N. The data are not publicly available due to the privacy of research participants. All authors have read and agreed to the published version of the manuscript.

## Supplementary Materials

The following supporting information can be downloaded at: https://www.mdpi.com/article/10.3390/jox15040107/s1, Figure S1 Superimposed chromatograms of blank PBMCs and LLOQ were GS-443902 had a nominal concentration of 0.313ng/sample; Table S1 Mean values (CI90) comparison of GS-443902 levels in PBMC with plasma GS-441524 and RDV

## Abbreviations

The following abbreviations are used in this manuscript:

SARS-CoV-2	Severe Acute Respiratory Syndrome Coronavirus Type 2
WHO	World Health Organization
RDV	Remdesivir
PK	Pharmacokinetics
PBMCs	peripheral blood mononuclear cells
HPLC-MS/MS	High-Performance Liquid Chromatography coupled with tandem Mass Spectrometry
STD 9	highest calibrator standard
RSD	relative standard deviation
IS	Internal Standard
QC	Quality Control
LLOQ	Lower Limit of Quantitation
RT	retention time
LOD	limit of detection
STD1	lowest calibrator standard
REC	Recovery
ME	matrix effect
TFV-d6	^ 2 ^ H_6_-tenofovir diphosphate
SPE	solid phase extraction
TDF	Tenofovir disoproxil fumarate
TAF	Tenofovir alafenamide
ACN	Acetonitrile
MeOH	Methanol
DMSO	dimethyl sulfoxide
MCV	mean cell volume
