# Supplementary material for: A Novel HPLC-MS/MS Method for the Intracellular Quantification of the Active Triphosphate Metabolite of Remdesivir: GS-443902
Source: J Xenobiot. 2025 Jul 3;15(4):107. doi: 10.3390/jox15040107 (PMC12285933; doi:10.3390/jox15040107)
Supplement: Supplementary file 1 [file jox-15-00107-s001.zip › jox-3570628-supplementary.pdf]

Journal of Xenobiotics

MDPI

Supplementary Materials: A Novel HPLC-MS/MS Method for the Intracellular Quantification of the Active Triphosphate Metabolite of Remdesivir: GS-443902

Alice Palermiti, Amedeo De Nicolò, Miriam Antonucci, Sara Soloperto, Martina Billi, Alessandra Manca, Jessica Cusato, Giorgia Menegatti, Mohammed Lamorde, Andrea Calcagno, Catriona Waitt and Antonio D'Avolio

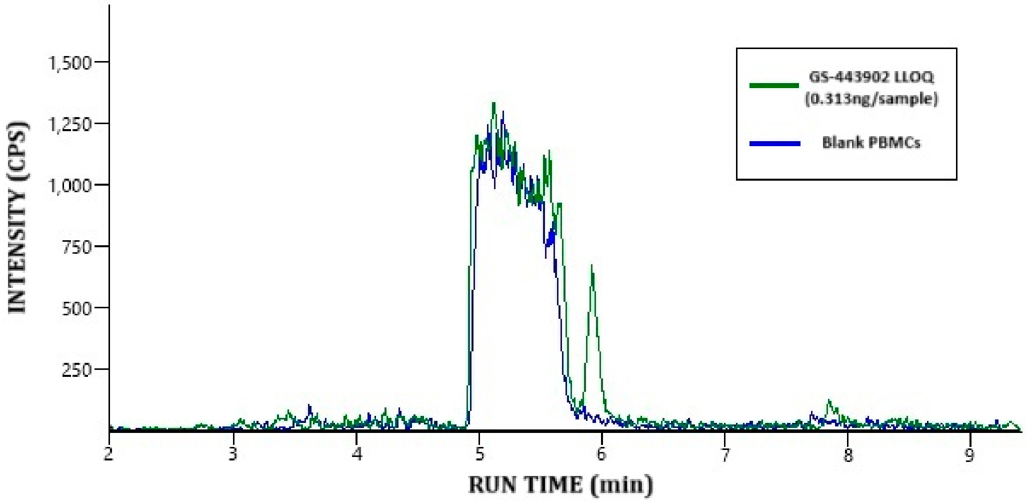

Figure S1. Superimposed chromatograms of blank PBMCs and LLOQ were GS-443902 had a nominal concentration of 0.313ng/sample.

Table S1. Mean values (CI_{90}) comparison of GS-443902 levels in PBMC with plasma GS-441524 and RDV.

	Mean Values (CI_{90})			
**Timing**	**RDV (ng/mL)**	**GS-441524 (ng/mL)**	**GS-443902 (ng/mL)**	**GS-443902 (µM)**
**Ctrough**	n.d. (n.d. – n.d.)	75.4 (28.1 – 122.7)	4853 (2232 – 7474)	9.15 (4.21 – 14.10)
**Cmax**	2255 (628 – 3883)	100.2 (44.1 – 156.3)	10735 (5802 – 15668)	20.25 (10.95 – 29.56)
**C1 hour post-dose**	307 (38 – 576)	104.0 (56.4 – 151.6)	9140 (5164 – 13116)	17.25 (9.74 – 24-74)
